# Occupational Exposure in Industrial Painters: Sensitive and Noninvasive Biomarkers to Evaluate Early Cytotoxicity, Genotoxicity and Oxidative Stress

**DOI:** 10.3390/ijerph18094645

**Published:** 2021-04-27

**Authors:** Delia Cavallo, Cinzia Lucia Ursini, Anna Maria Fresegna, Aureliano Ciervo, Raffaele Maiello, Giuliana Buresti, Enrico Paci, Daniela Pigini, Monica Gherardi, Damiano Carbonari, Renata Sisto, Giovanna Tranfo, Sergio Iavicoli

**Affiliations:** Department of Occupational and Environmental Medicine, Epidemiology and Hygiene, Italian Workers’ Compensation Authority—INAIL, Monte Porzio Catone, 00078 Rome, Italy; c.ursini@inail.it (C.L.U.); a.fresegna@inail.it (A.M.F.); au.ciervo@inail.it (A.C.); r.maiello@inail.it (R.M.); g.buresti@inail.it (G.B.); e.paci@inail.it (E.P.); d.pigini@inail.it (D.P.); m.gherardi@inail.it (M.G.); d.carbonari@inail.it (D.C.); r.sisto@inail.it (R.S.); g.tranfo@inail.it (G.T.); s.iavicoli@inail.it (S.I.)

**Keywords:** occupational VOCs exposure, painters, biological monitoring, BMCyt assay, Fpg comet assay, oxidative stress biomarkers, proinflammatory effects

## Abstract

This study aimed to identify sensitive and noninvasive biomarkers of early cyto-genotoxic, oxidative and inflammatory effects for exposure to volatile organic compounds (VOCs) in shipyard painters. On 17 (11 spray and 6 roller) painters (previously characterized for VOCs exposure to toluene, xylenes, ethylbenzene, ethyl acetate) and on 18 controls, we performed buccal micronucleus cytome (BMCyt) assay; Fpg-comet assay on lymphocytes; detection of urinary 8-oxoGua (8-oxo-7,8-dihydroguanine), 8-oxodGuo (8-oxo-7,8-dihydro-2′-deoxyguanosine) and 8-oxoGuo (8-oxo-7,8-dihydroguanosine), and cytokines release on serum. We found induction of cyto-genotoxicity by BMCyt assay and inflammatory effects (IL-6 and TNFα) in roller painters exposed to lower VOC concentrations than spray painters. In contrast, in both worker groups, we found direct and oxidative DNA damage by comet assay (with slightly higher oxidative DNA damage in roller) and significant increase of 8-oxoGuo and decrease of 8-oxodGuo and 8-oxoGua in respect to controls. The cyto-genotoxicity observed only on buccal cells of roller painters could be related to the task’s specificity and the different used protective equipment. Although limited by the small number of subjects, the study shows the usefulness of all the used biomarkers in the risk assessment of painters workers exposed to complex mixtures.

## 1. Introduction

Volatile organic compounds (VOCs) are present in many products and used in several working activities. Furthermore, human beings emit many volatile organic compounds produced by endogenous metabolism depending on dietary and smoking habits, activity level, state of health, environmental exposures, age, stress level, or mood. The most abundant compounds are CO_2_, acetone, and acetaldehyde [1]. However, the continuous exposure to some VOCs is hazardous, as many of them have been classified as carcinogenic by International Agency for Research on Cancer IARC [2] with consequent potential adverse effects on human health. Organic solvents easily evaporate at room temperature, spread in the environment, and could induce toxic effects, particularly in the occupational field, where possible exposure could be relevant. Particularly in occupational settings, VOCs must be handled carefully using safety precautions, such as wearing personal protection equipment to avoid or minimize exposure. Occupational exposure assessment to airborne chemicals in workplaces is carried out once a year, or even less frequently, as it is a great organizational and economical effort for the employer. Validated methods for measuring exposure to chemical agents use static samplers and analytical techniques that provide an offline and time-averaged response. Current advances in developing low-cost microscale sensing technology, if sufficiently accurate for evaluating the exposure or toxicological studies, could help ensure the protection of workers’ health. High-performance sensors to detect many different gases and VOCs have been developed in recent years, such as high porosity MOF sensors [3,4].

VOC exposure can induce respiratory, immunologic, carcinogenic, reproductive, cardiovascular and inflammatory effects [5,6]. One of the main effects induced by VOCs on humans is the damage on nucleic acids producing oxidative stress, genotoxicity and inflammation [7,8].

In the occupational field, effect biomarkers will permit us to measure the health effect of occupational exposures, even in conditions that are regarded as not dangerous, when workers’ exposure values are below the occupational exposure limits, like in the case of chronic exposure to very low levels of xenobiotics, or in the case of exposure to mixtures of several substances [9].

Painters represent a worker category significantly exposed to VOCs. The IARC classified occupational exposure to paints as a high-risk group for lung and bladder cancer development [10], with the main pathways of exposure involving inhalation of vapors and gases, dermal absorption and/or ingestion. Painting activity is giving work to more than 16,000 employees only in Italy. Several studies evaluated the potential adverse effects of exposure to toluene, xylenes, ethylbenzene, styrene and paints in painters, and they showed mainly oxidative and genotoxic effects [6,11,12,13,14,15]. In particular, DNA damage by comet assay, but not micronuclei induction was found by Moro et al. [12] on lymphocytes of industrial painters exposed to low toluene levels. Induction of DNA damage on buccal cells and lymphocytes by comet assay, but not induction of micronuclei on buccal cells were found on Brazilian paint industry workers by Oliveira et al. [16]. Chang et al. [17] reported a significant correlation between urinary 8-oxo-7,8-dihydro-2′-deoxyguanosine (8-OHdG) and exposure to ethylbenzene in spray painters. The exposure to organic solvents and paints resulted also associated with oxidative DNA damage evaluated by formamidopyrimidine-glycosylase (Fpg) comet assay on lymphocytes of car painters [15]. Celik et al. [13] found micronucleus induction on exfoliated buccal cells of Turkish painters. Another recent study using buccal micronucleus cytome (BMCyt) assay found either cytotoxic or genotoxic effects on buccal cells of Brazilian car painters [18].

The biomonitoring of workers exposed to VOCs, using biomarkers of exposure and of early genotoxic/oxidative and inflammatory effects, can provide useful tools for early detection of toxicity. In addition, it can play an important role in predicting health risks helping to prevent developing diseases [12,13,19].

In the present study, we evaluated in a group of 17 shipyard painters (11 spray and 6 roller) early cyto-genotoxic and oxidative-inflammatory effects by specific effect biomarkers and correlated them with VOCs airborne concentrations and VOCs urinary metabolites detected in a previously published study on the same workers [20]. The aim was to identify suitable, sensitive and noninvasive biomarkers of early cyto-genotoxic, oxidative and inflammatory effects for the biomonitoring of painters exposed to complex mixtures, including mainly VOCs at relatively low doses.

For this purpose, we employed: the noninvasive BMCyt assay, a biomarker of cyto-genotoxicity at the level of buccal mucosa; Fpg modified Comet assay, a sensitive biomarker of early direct/oxidative DNA damage on lymphocytes [21]; urinary oxidized nucleic acid bases 8-oxo-7,8-dihydroguanine (8-oxoGua), 8-oxo-7,8-dihydro-2′-deoxyguanosine (8-oxodGuo) and 8-oxo-7,8-dihydroguanosine (8-oxoGuo), sensitive biomarkers of oxidative stress; serum proinflammatory (IL-6, IL-8, TNFα) cytokine detection, markers of early inflammation. We chose BMCyt assay because the buccal cells are the specific target organ of inhalation exposure, so it could be a suitable biomarker of cyto-genotoxicity for the VOCs exposed workers, as shown in our previous study on workers exposed to styrene [22]. The oxidative stress was determined using different effect biomarkers, such as the detection of direct oxidation products generated from the DNA and RNA repair and turnover and oxidative DNA damage evaluated by Fpg-comet assay.

## 2. Materials and Methods

### 2.1. Subjects and Study Design

The study was performed on 17 shipyard painters (all males) exposed to organic solvents (toluene, xylene, ethylbenzene, etc.) contained in paints and to other substances, like diluents and additives (ethyl acetate, n-butyl acetate). Eighteen healthy subjects non-occupationally exposed with mean age, smoking habit and sex comparable to those of exposed subjects were selected as controls. All enrolled subjects participating in the study gave their written informed consent and filled a questionnaire, including age, gender, smoking habit, general health status, professional task and exposure to organic solvents, job seniority and use of personal protective equipment.

This study followed the Declaration of Helsinki and was performed following the ethical standards of our Institutional Committee and following the approval of the local health agency of the Region Marche.

Among the enrolled shipyard painters, we identified two working tasks, roller- and spray-painting, that included 6 spray- and 11 roller-painters, using full-facepiece and half-face piece respirators with carbon filters, respectively.

Personal exposure to organic solvents and biological monitoring results on the same workers have been previously published [20,23]. In particular, airborne ethyl acetate, n-butyl-acetate, o-xylene, m-xylene, p-xylene, toluene, ethylbenzene were measured during the whole work-shift and the urinary metabolites of xylenes (2,3 and 4 methyl hippuric acids—MHIPP), toluene (S-benzyl mercapturic acid—SBMA) and ethylbenzene (phenylglyoxylic and mandelic acid—PGA and MA) were measured in the urine before and after the work shift.

**Figure 1:**
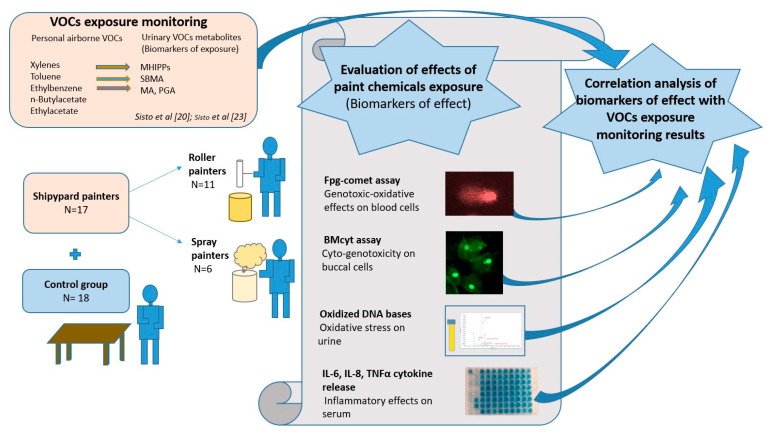
Study design.

In the present study, we evaluated cyto-genotoxic, oxidative and inflammatory effects of painters, comparing them with those of a control group of unexposed subjects considering either the painters all together or each painter group (roller and spray) according to the specific task. In addition, we correlated the observed effects in exposed workers with the previously published VOC exposure monitoring data [20,23]. The study design details are reported in Figure 1.

### 2.2. Buccal Micronucleus Cytome (BMCyt) Assay

We carried out the buccal cells sampling at the start of the working shift on a Wednesday (third working day). The studied subjects collected exfoliated buccal cells by gently scraping the internal mucosa of the cheeks with a wet toothbrush (previously immersed in phosphate buffer solution–PBS) and after having washed out the mouth with water. The obtained cells were suspended in 25 mL of Titenko-Holland buffer solution containing 0.01 M Tris-HCl, 0.1 M EDTA and 0.02 M NaCl (pH 7) and moved to the laboratory where BMCyt assay was performed. The buccal cells were washed twice in Titenko-Holland buffer solution, and 50 µL of the obtained cell suspension (1.5 × 10^6^–2 × 10^6^/mL) were dropped on prewarmed slides (37 °C). Slides were air-dried and fixed in 80% methanol for 48 h, then, we stained the cells with orange acridine (0.005%, Sigma) and observed them by fluorescence microscope at 400× magnification (Leica, Germany). For each subject, two expert readers analyzed at least 2000 differentiated cells according to the criterion established by Titenko-Holland et al. [24]. We recorded separately such events: the presence of cells with micronucleus (MN), nuclear buds (NB) and broken eggs (BE), all indicative of DNA damage; of binucleated cells (BIN), indicative of cytokinesis defect or arrest; of karyolytic cells (KL), indicative of the advanced stage of necrosis and apoptosis and of cells with condensed chromatin (CC) indicative of early stages of apoptosis. On each subject, we evaluated the frequency of each abnormality on total differentiated exfoliated cells and expressed them as‰. Moreover, subjects with micronucleated cell frequency exceeding a fixed cutoff value (1.5‰) were identified as positive to MN assay. We chose 1.5‰ threshold based on the results of the human micronucleus project on exfoliated buccal cells (HUMNXL) published by Bonassi et al. [25] that report the estimated spontaneous MN frequency of 0.74‰ (95% CI 0.52–1.05) and, in particular, for our staining method (acridine orange) a mean value of 0.98‰ (95% CI 0.39–1.14). Then, we established a cutoff value of 1.5 above both the upper limits of confidence intervals.

### 2.3. Direct/Oxidative DNA Damage (Fpg-Comet Assay)

Blood sample collection was performed at the end-shift of the same Wednesday of buccal cells and urine sampling. Specialized medical personnel collected whole venous blood samples from exposed and control subjects by venipuncture in sterile heparinized disposable syringes and transferred them in the dark and at +4 °C to the laboratory where the assay was performed. We applied an Fpg-modified comet assay to measure direct and oxidative DNA damage. The Fpg is a glycosylase that recognizes and cuts the oxidized bases (principally 8-oxo-7,8-dihydroguanine) from DNA, generating apurinic sites converted in breaks by the associated AP-endonuclease activity. Such breaks are detected by comet assay as Fpg sites estimating oxidative DNA damage. Lymphocytes were isolated from whole blood on a Ficoll-based density gradient and suspended in 1 mL of PBS, then the procedure of Collins et al. [21], with minor modifications [26] was applied. For each sample, images of 100 randomly selected comets were acquired, and a specific image analyzer software (Delta Sistemi, Roma, Italy) was used to analyze them. We estimated the mean values of the three main and more used tail comet parameters: tail DNA%, tail length (TL) and tail moment (TM). Tail DNA% (ratio of tail intensity and total intensity of the comet) measures the number of broken pieces of DNA; TL measures the smallest detectable size of migrating DNA (small DNA fragments with high capacity to migrate); TM (product of the tail length and the percentage DNA in tail) takes into account both the above parameters. The combined use of the three parameters allows the evaluation of the capability of a genotoxic agent to break the DNA strands into fragments of different sizes.

Then, we measured these comet parameters from enzyme untreated cells to evaluate direct DNA damage for each subject.

Following Collins et al. [27] suggestions, we assessed oxidative DNA damage using the tail DNA% parameter since it provides the best estimate of the frequency of DNA breaks included those due to the Fpg enzyme (relative to oxidized DNA bases). We measured tail DNA% from Fpg-enzyme-treated cells (tail DNAenz%) (evaluating direct and oxidative DNA damage), and we deducted tail DNA% from the tail DNAenz% both in exposed and control subjects to obtain oxidative DNA damage (Fpg sites). Subjects with mean values of the difference (tail DNAenz%–tail DNA%) exceeding a fixed arbitrary cutoff value of 4 were identified as positive to oxidative DNA damage.

### 2.4. Urinary Oxidized Nucleic Acid Bases (8-oxoGua, 8-oxodGuo and 8-oxoGuo)

Urine samples were collected at the end-shift of the same Wednesday of blood and buccal cell sampling in sterile plastic containers, were divided into three aliquots in polypropylene screw-cap tubes and stored frozen at −20 °C until analysis. The urine samples were analyzed on a Series 200 LC quaternary pump (PerkinElmer, Norwalk, CT, USA), coupled with an AB/Sciex API 4000 triple-quadrupole mass spectrometry detector equipped with a turbo ion spray (TIS) probe.

The method described in Andreoli et al. [28] with some modifications [29] was applied to the urine samples to assess the concentrations of 8-oxoGua, 8-oxodGuo, 8-oxoGuo and their internal standards, ((^13^C^15^N_2_) 8-oxoGua), ((^13^C^15^N_2_) 8-oxodGuo) and ((^13^C^15^N_2_) 8-oxoGuo), using the isotopic dilution method. The 1.5 version of Analyst® software (AB Sciex, Framingham, MA, USA) was employed for instrument control.

To normalize the results for the dilution grade of urine, the results were expressed as the ratio to the concentration of urinary creatinine. The method of Jaffe using alkaline picrate test with UV-vis detection at 490 nm [30] was applied to determine the urinary creatinine. Samples with creatinine concentrations lower than 0.3 g/L or higher than 3.0 g/L were excluded from statistical analysis according to the American Conference of Governmental Industrial Hygienists (ACGIH) recommendation [31].

### 2.5. Cytokine Release

We evaluated in the enrolled subjects, the release of proinflammatory cytokines TNFα, IL-6 and IL-8 on the serum obtained from whole blood by separation on a Ficoll-based density gradient and stored at −80 °C until use. Cytokines concentrations were detected by human enzyme-linked immunosorbent assay (ELISA) eBioscience assay kits (Vienna, Austria) according to the manufacturer’s guidelines. The absorbance was measured at 450 nm and quantified with a microplate reader (iMark, Bio-Rad, Milan, Italy). The limits of detection (LOD) were 0.92 pg/mL for IL-6, 2.00 pg/mL for IL-8, 2.3 pg/mL for TNFα.

### 2.6. Statistical Analysis

Statistical analysis was performed with IBM SPSS software version 25 (IBM, Armonk, NY, USA). Chi-squared test and Fisher’s exact test were used to test the significance of the association between categorical variables and groups analyzed. A preliminary test of normality of parameter distributions was performed to establish the type of test required to compare mean values in the groups of exposed and controls. Shapiro-Wilks test for normality was considered in the analysis. One-way ANOVA and Student’s *t*-test were used in case of normal distribution of parameters; while Kruskal–Wallis and Mann–Whitney nonparametric tests were used to test the significance of mean values differences between controls and exposed subgroups/group in case of non-Gaussian distribution. Pair-wise comparisons were performed using Bonferroni test and Dunn’s procedure with a Bonferroni correction for multiple comparisons. The correlation between two variables was determined by Pearson’s correlation coefficients. A *p* value < 0.05 was considered statistically significant.

## 3. Results

The characteristics of the studied workers and controls are reported in Table 1.

Statistical analysis of exposed workers and controls for age and smoking habit did not show any statistically significant difference; in contrast, there was a statistically significant difference for job seniority in the specific task since spray painters were characterized by more years of employment (Table 1). Therefore, we performed a regression analysis of task seniority and specificity of the task in exposed groups (roller/spray) on biomarker of effect and did not find any influence of task seniority variable on such biomarkers, neither singularly nor in the interaction with the type of task.

Table 2 shows BMcyt assay results either taking into account all the exposed workers concerning controls or comparing the two painters groups (roller and spray) with each other and with controls.

When the painters were considered altogether, we found statistically significantly higher frequencies of all the tested parameters compared to controls. Kruskal–Wallis multiple comparisons of roller painters, spray painters and controls showed statistically significant higher genotoxic effects in terms of MN and nuclear bud frequencies in roller painters concerning controls (Table 2).

We also found 23.5% of MN-positive subjects in all painters taken all together, who belonged all to the roller group with 36.4% of MN positivity and a lack of MN-positive subjects in spray painters and controls. A significantly higher KL cell frequency, indicative of late apoptosis and cell death, was also found for roller painters compared to controls, while an increase of cells with condensed chromatin, indicative of early apoptosis, was found in both painters populations.

*t*-Test and Mann–Whitney statistical analyses performed on exposed workers and controls to verify the confounding factor smoking habits on BMcyt results did not show significantly higher frequencies of analyzed parameters in smoking exposed subjects.

Fpg-comet assay results are shown in Table 3, where the data are reported either taking into account the exposed workers all together compared to controls or the two painters groups (roller and spray) compared each other and with controls. The painters considered all together showed a statistically significant increase of tail DNA%, TM and TL mean values (indicative of direct DNA damage) concerning controls. Taking into account roller painters, spray painters and controls, we found statistically significant higher tail DNA%, TM and TL mean values in both groups of exposed workers than controls. Furthermore, the percentages of comets and apoptotic cells were significantly higher in both groups of painters than in controls.

The mean values of the oxidative DNA damage parameter (Fpg sites relative to tail DNA%) were higher in both exposed groups concerning controls reaching the statistical significance in roller painters and in exposed workers taken all together (Table 3). The subjects with oxidative DNA damage were 54.0% in roller and 50.0% in spray painters concerning 16.7% of the control group. *t*-Test and Mann–Whitney statistical analyses performed on exposed workers and controls to verify the confounding factor smoking habit on Fpg-comet results did not show any statistical association with the smoking factor, excluding the influence of this factor on the observed results.

Table 4 and Table 5 show the Pearson’s correlation analysis performed among BMcyt and Fpg-comet assay results with urinary VOC metabolite concentrations evaluated, taking into account all the exposed painters (Table 4) and the specific task (Table 5).

A positive correlation between CC cell frequency and the ethylbenzene metabolite PGA was found either for exposed workers taken into account all together (Table 4) or for roller painters (Table 5). Whereas, in spray painters, we found a positive correlation between KL cell frequency and the xylene metabolite 2-MHIPP (Table 5).

Fpg-comet results and urinary VOC metabolites in painters taken all together showed a positive statistically significant correlation between the urinary toluene metabolite SBMA and direct DNA damage parameters TM and tail DNA% (Table 4).

When we considered the specific task, the positive correlation between SBMA and DNA damage parameters (TM and TL) was found only in the roller group (who however was almost two-fold against spray painters) and increased (Table 5). Then we can hypothesize that the positive correlation found in painters taken all together was due to the more numerous roller group. In spray painters, negative correlations of TM with ethylbenzene metabolites (MA and PGA) and xylene metabolite 2-MHIPP were found. In addition, this last metabolite (2-MHIPP) was also negatively correlated with TL.

Pearson’s correlation analysis performed among BMcyt and Fpg-comet assay results with airborne VOCs showed statistical significance only in spray workers reported in Table 6. We found a positive correlation of KL cell frequency with n-butyl acetate and particularly with o-xylene. In fact, personal airborne o-xylene concentration in spray painters was higher (22.82 vs. 2.52 mg/m^3^) than in roller painters (*t*-test *p* value = 0.009) [20].

The correlation between Fpg-comet results and personal airborne VOCs showed a strong negative correlation between o-xylene exposure and TM and TL (Table 6).

The results relative to urinary biomarkers of oxidative stress, partially (those relative to exposed subjects) previously published [20,23], and those regarding biomarkers of inflammation are reported in Table 7. In this Table a significant increase of urinary 8-oxoGuo and a decrease of 8-oxodGuo and 8-oxoGua are shown in both painters groups concerning controls and in painters taken all together.

Regarding cytokine release, the table shows the cytokine concentrations of subjects with levels higher than LOD. In both painters groups we found the highest IL-8 values in subjects resulted also positive to oxidative DNA damage by Fpg-comet. Moreover the only subject with detectable levels of IL6 and TNFα was found in the roller group and was also positive to oxidative DNA damage.

The table also shows the percentages of subjects with detectable levels of cytokine. In both painters groups, the percentages relative to IL-8 were slightly higher than in controls, whereas subjects with detectable levels of IL6 and TNFα were found only in the roller group.

No correlation between urinary excretion of oxidative DNA bases and oxidative DNA damage was found by Pearson’s correlation analysis, neither taking into account painters all together nor in relation to the specific task.

## 4. Discussion

The combined exposure to multiple chemicals raises concerns about the effects on health, as the failure to account for the effects of combined exposures could lead to underestimation of risk. The current prevention practices are largely based on considering single chemical substances. Multiple exposures and their combined effects require better management to protect occupational and public health and the environment from hazardous chemical mixtures [32].

In this study, several biomarkers of cyto-genotoxicity, oxidative and inflammatory effects, such as BMcyt and Fpg-comet assays, urinary oxidative DNA bases and cytokine release detection, were applied to painters, who represent a category of workers exposed to complex chemical mixtures. The painters were previously characterized for VOC exposure, with values of potential inhalation exposure to each solvent below the relative Occupational Exposure Limits, and toluene and xylenes resulted at the highest concentrations in the mixture [20,23].

The two groups of studied painters, characterized by the specific roller and spray task, differed in the VOCs exposure, with spray painters exposed to higher levels of airborne toluene, xylenes, ethylbenzene and ethyl acetate concerning roller painters [20]. The urinary concentrations of the relative VOCs metabolites measured at the end of the work shift showed for the xylene metabolite 3 + 4 MIHPP a significantly higher level in spray than in roller painters [16].

However, in both painters groups, the values of xylenes, toluene and ethylbenzene urinary metabolites were all well below the biological exposure indexes (BEI) given by the ACGIH. Although the exposure levels are quite close to the threshold limit value (TLV) for the mixture regarding the airborne concentration, in the case of the spray painters, the personal protective equipment can effectively reduce the internal exposure [23].

The noninvasive and sensitive BMCyt assay demonstrated genotoxicity at the target organ, evidencing a statistically significant increase of micronucleated cells and the presence of MN-positive subjects together with the increase of cells with nuclear buds in roller painters. The increase of karyolitic cells and of cells with condensed chromatin, indicative of early apoptosis and cell death observed in all painters, suggests that exposure to xylenes, toluene and ethylbenzene can also induce cytotoxic effects, as previously shown for styrene [18]. In particular, in spray painters, the correlation of karyolitic cell frequency with o-xylene exposure agrees with that found with its metabolite 2-MHIPP, while in roller painters, the condensed chromatin cell frequency was correlated with the ethylbenzene metabolite PGA.

BMcyt assay also showed in the spray group, characterized not only by higher environmental VOC exposure level but also by higher job seniority, a negative correlation, even if not statistically significant, between genotoxic parameters (MN frequency and nuclear buds) and xylenes, ethylbenzene, n-butyl acetate exposure (level and duration) suggesting higher DNA repair efficiency in workers chronically exposed to moderate VOCs levels. This latter hypothesis could also explain the statistically significant negative correlation found in the most and longest exposed spray painters between o-xylene exposure and direct DNA damage evaluated by comet assay.

The genotoxicity observed on buccal cells of roller painters could also be explained by the specificity of the task, especially to using different protective equipment, as they wore half face-piece respirators, while spray painters had full face-piece respirators, and to the different paints used, containing chemicals other than those measured.

The direct DNA damage found by comet assay in both working groups and the significant association between tail DNA% and TM biomarkers with SBMA (a specific metabolite of toluene), found in roller painters (who however was almost two-fold against spray painters), suggest that toluene could directly interact with DNA. These findings seem to confirm those found by Moro et al. [12], who demonstrated the presence of direct DNA damage, evaluated by comet assay, in painters exposed to relatively low toluene levels. The same authors in a previous study showed increased levels of oxidative stress biomarkers malondialdehyde (MDA), superoxie dismutase (SOD) and catalase (CAT) in industrial painters exposed to toluene, xylene, styrene, ethylbenzene and lead, suggesting toluene as the principal factor responsible for increased lipid peroxidation [11]. An increase of oxidative DNA injury in occupational exposure to paint was reported by Chang et al. [17], who showed in spray painters a significant correlation between urinary 8-OHdG and exposure to ethylbenzene. Londono-Velasco et al. [15] found an association between exposure of car painters to organic solvents and paints and increased oxidative DNA damage evaluated by Fpg-comet assay on lymphocytes.

The induction of oxidative DNA damage assessed by Fpg-comet (either in terms of increased Fpg sites mean values or percentages of subjects with oxidative DNA damage) observed in the present study on both painter groups, exposed to relatively low levels of VOCs (including toluene, xylenes, ethylbenzene), seems to confirm the above-cited studies. We also found statistically significant higher urinary 8-oxoGuo (related to the RNA oxidation) levels in both painter groups compared with controls, whereas 8-oxoGua and 8-oxodGuo, coming from the DNA repair and turnover, were significantly decreased. The higher excretion of 8-oxoGuo could be related to the higher susceptibility of RNA to oxidative insults, in fact, RNA is single-stranded, and its bases are not protected by hydrogen bonds or structural proteins and may be more susceptible to oxidative insults than DNA [33], for which efficient repair systems (Base Excision Repair and Nucleotide Excision Repair) are available. These results are also in agreement with Tranfo et al. [29], where 8-oxoGuo is highlighted as the most suitable among the nucleic acid oxidation biomarkers to study the effect of low-level chemical exposure. The lower excretion of 8-oxoGua and 8-oxodGuo and the simultaneous presence of oxidative DNA damage observed by Fpg-comet assay could be attributed to incomplete DNA repair due to saturation or inefficiency of DNA-repair systems.

Finally, the inflammatory effects that we found in terms of IL-8 release in both painters groups and of IL-6, and TNFα releases in roller painters were also associated with the presence of oxidative DNA damage. Significant changes in inflammatory parameters (cytokines and nitric oxide levels) and in the DNA damage marker 8-oxo-7,8-dihydro-2′-deoxyguanosine in painters were also reported in a recent study [34].

Lack of MN induction in buccal cells, but presence of genotoxicity in blood cells detected by comet assay in the more VOCs exposed spray painters found in this study, has been already observed in a population of car painters by de Oliveira et al. [16], who also found genotoxicity by comet only on buccal cells. The authors explained that positive results in the comet assay do not always correspond to positive results in the MN test, especially when the exposure to genotoxic agents is low. The comet assay usually detects more DNA defects than the MN test. The genotoxic effects detected by the comet assay and MN tests are due to different mechanisms; the MN test detects damages that survive at least one mitotic cycle, while the comet assay identifies repairable DNA strand breaks or alkali-label sites, so using both the MN test and the comet assay is advisable [16].

## 5. Conclusions

Our findings, although limited by the small number of studied subjects, demonstrate the usefulness of all the used biomarkers, and particularly the good performances of the BMcyt assay (the most suitable in the case of inhalation exposure), that can detect not only genotoxicity but also cytotoxicity using noninvasive sampling. In fact, this assay allowed us to detect cytotoxic effects in both painters’ groups, and genotoxicity, expressed in terms of micronuclei and nuclear buds induction, only in the fewer VOCs exposed roller painters, suggesting as probable cause a different and task-related exposure. Fpg-comet results showed on both painters’ groups direct and oxidative DNA damage induction, confirming the very high sensitivity of this assay in its capability to detect early and still repairable DNA damage, including oxidative effects, together with the detection of oxidized DNA bases in the urine, particularly 8-oxoGuo. These assays demonstrated to be, among the biomarkers used to study genotoxic and oxidative effects of moderate exposure to VOCs in industrial painters, the most sensitive ones, showing for direct DNA damage a positive correlation with the toluene metabolite SBMA. The study shows that chronic occupational exposure to relatively low concentrations of VOCs and paints may lead to increased risk of cytotoxicity at target organ for inhalation exposure and genetic damage among paint industry workers. It suggests to use simultaneously all the mentioned biomarkers for the risk assessment of this category of workers, exposed to complex mixtures of chemicals. However, further studies on larger worker populations are needed to confirm such results.

The use of biomarkers evaluating different endpoints is particularly relevant and useful in the case of multiple exposures to chemicals because it allows highlighting the combined effects due to the different substances present in the mixture, also acting in a synergistic way, contributing to the correct and complete risk assessment and management.

## Figures and Tables

**Table 1:** Characteristics of the studied populations.

Characteristics	Roller Painters (N = 11) Mean ± SD	Spray Painters (N = 6) Mean ± SD	All Workers (N = 17) Mean ± SD	Controls (N = 18) Mean ± SD	*p* Value
Age (years)	36.00 ± 7.74	44.67 ± 5.90	39.05 ± 8.16	40.33 ± 12.49	(a) T Student’s = 0.722 (b) ANOVA = 0.255
Smoking habit					
Yes	2/11 (18.2%)	3/6 (50.0%)	5/17 (29.4%)	6/18 (33.3%)	(a) χ^2^ = 0.803; (b) χ^2^ = 0.389
no	9/11 (81.8%)	3/6 (50.0%)	12/17 (70.6%)	2/18 (66.7%)	
Task seniority (years)	8.26 ± 8.90	14.7 ± 5.90	10.52 ± 8.42		Spray vs. roller Mann–Whitney = 0.027

(a) exposed vs. controls; (b) specific task vs. controls.

**Table 2:** Buccal micronucleus cytome assay results.

Variable	Controls (N = 18)	Roller Painters (N = 11)	Spray Painters (N = 6)	All Painters (N = 17)
	Mean ± SD	Mean ± SD	Mean ± SD	Mean ± SD
**Genotoxicity**				
MN‰	0.05 ± 0.15	1.24 ± 0.92 **	0.23 ± 0.25	0.89 ± 0.90 °
NB‰	0.22 ± 0.30	1.25 ± 1.00 *	0.82 ± 0.53	1.1 ± 0.88 °
MN + NB‰	0.27 ± 0.34	3.62 ± 2.00 **	1.32 ± 1.03	2.81 ± 2.03 °°
MN-positive subjects	0/18 (0.0%)	4/11 (36.4%)	0/6 (0.0%)	4/17 (23.5%)
**Cytotoxicity**				
BIN‰	8.44 ± 9.25	11.46 ± 7.64	7.96 ± 2.96	10.23 ± 6.50 °
KL‰	40.44 ± 35.39	94.68 ± 35.39 *	88.65 ± 47.21	92.55 ± 38.58 °
CC‰	0.21 ± 0.37	2.78 ± 3.14 *	2.48 ± 1.70 *	2.67 ± 2.66 °°

MN—micronucleus; NB—nuclear buds; BIN—binucleated cells; KL karyolytic cells; CC—condensed chromatin. Kruskal–Wallis test followed by post hoc Dunn’s procedure * *p* < 0.05; ** *p* < 0.001 vs. controls; Mann–Whitney ° *p* < 0.05, °° *p* < 0.001 vs. controls.

**Table 3:** Fpg-comet assay results.

Variable	Controls (N = 18)	Roller Painters (N = 11)	Spray Painters (N = 6)	All Painters (N = 17)
	Mean ± SD	Mean ± SD	Mean ± SD	Mean ± SD
**Direct DNA damage**				
Tail DNA%	11.56 ± 2.62	17.42 ± 4.74 *	18.17 ± 3.88 *	17.68 ± 4.35 ^##^
TM	2.92 ± 1.02	5.58 ± 1.71 °	5.83 ± 2.0 °	5.67 ± 1.76 ^^
TL	17.65 ± 8.33	26.94 ± 4.68 °	26.69 ± 10.73	26.85 ± 7.05 ^
**Oxidative DNA damage**				
Fpg sites (tail DNA%)	2.35 ± 1.47	4.93 ± 3.53 *	4.31 ± 2.24	4.71 ± 3.07 ^#^
Positive subjects	3/18 (16.7%)	6/11 (54.5%)	3/6 (50.0%)	9/17 (53.0%)

TM—tail moment; TL—tail length. Data relative to comet parameters of “All painters” group are previously published in [23]. * ANOVA test. Multiple comparison: Bonferroni test (adjusted *p*-value < 0.005) vs. controls, ° Kruskal–Wallis test. Multiple comparison followed by Dunn’s procedure adjusted *p*-value < 0.05 vs. controls; T—Student’s test: ^#^ *p* < 0.05; ^##^ *p* < 0.001 vs. controls; Mann–Whitney test: ^ *p* < 0.05; ^^ *p* < 0.001 vs. controls.

**Table 4:** Pearson’s correlation analysis between biomarkers of exposure and biomarkers of effect evaluated, taking into account all the exposed painters.

		MN‰	NB‰	(MN + NB)‰	BIN‰	KL‰	CC‰	TM	Tail DNA%	TL	Fpg Sites Tail DNA%
MA	r	0.041	−0.344	−0.015	−0.122	0.050	0.087	−0.032	0.154	−0.079	−0.132
	*p*	0.877	0.176	0.954	0.641	0.848	0.741	0.904	0.554	0.764	0.613
PGA	r	−0.009	0.175	0.211	0.271	0.290	0.600 *	−0.039	0.072	−0.132	0.148
	*p*	0.972	0.502	0.417	0.292	0.259	0.011	0.882	0.783	0.613	0.570
2-MHIPP	r	0.011	−0.246	−0.040	−0.090	0.037	0.033	0.058	0.222	−0.108	−0.136
	*p*	0.966	0.341	0.880	0.732	0.887	0.900	0.826	0.391	0.681	0.604
3+4 MHIPP	r	−0.315	−0.300	−0.340	−0.172	0.034	0.116	0.040	0.180	−0.107	0.045
	*p*	0.219	0.242	0.182	0.509	0.897	0.659	0.878	0.488	0.684	0.865
SBMA	r	0.262	−0.047	0.213	0.250	0.094	−0.111	0.491 *	0.548 *	0.295	−0.001
	*p*	0.310	0.857	0.411	0.334	0.719	0.673	0.045	0.023	0.251	0.996

Bold indicates statistically significant values. MA—mandelic acid; PGA—phenylglyoxylic acid; SPMA—S-phenylmercapturic acid; 2-MHIPP—2-methylhippuric acid, 3+4 MHIPP—3 and 4-methylhippuric acid; SBMA—S-benzyl mercapturic acid; MN—micronucleus; NB—nuclear buds; BIN—binucleated cells; KL—karyolytic cells; CC—condensed chromatin; TM—tail moment; TL—tail length. * The correlation is significant at 0.05—two tails.

**Table 5:** Pearson’s correlation analysis between urinary biomarkers of exposure and biomarkers of effect in the two groups of painters.

Metabolite		MN‰	NB‰	MN + NB‰	BIN‰	KL‰	CC‰	TM	Tail DNA%	TL	Fpg-Sites Tail DNA%
**Roller**											
MA	r	0.213	−0.266	0.212	−0.119	−0.239	−0.016	0.384	0.384	0.430	−0.189
	*p*	0.529	0.430	0.532	0.727	0.479	0.964	0.243	0.244	0.187	0.577
PGA	r	0.022	0.237	0.338	0.277	0.184	0.632 *	0.226	0.198	0.255	0.140
	*p*	0.950	0.483	0.309	0.410	0.588	0.037	0.504	0.560	0.449	0.682
2-MHIPP	r	0.239	−0.214	0.183	−0.106	−0.298	−0.039	0.430	0.381	0.492	−0.154
	*p*	0.478	0.528	0.590	0.756	0.374	0.909	0.187	0.247	0.124	0.652
3 + 4 MHIPP	r	0.043	−0.137	0.060	−0.028	−0.120	0.146	0.406	0.333	0.386	0.126
	*p*	0.900	0.689	0.860	0.936	0.725	0.669	0.216	0.318	0.241	0.713
SBMA	r	0.476	-0.169	0.334	0.445	0.356	0.067	0.749 **	0.779 **	0.572	−0.124
	*p*	0.139	0.619	0.316	0.170	0.283	0.845	0.008	0.005	0.066	0.715
**Spray**											
MA	r	−0.507	−0.266	−0.596	−0.005	0.547	0.539	−0.823 *	−0.484	−0.573	0.099
	*p*	0.305	0.430	0.212	0.993	0.261	0.270	0.044	0.331	0.235	0.853
PGA	r	−0.223	0.237	−0.194	0.405	0.651	0.413	−0.837 *	−0.466	−0.797	0.216
	*p*	0.672	0.483	0.713	0.426	0.161	0.415	0.038	0.352	0.057	0.681
2-MHIPP	r	−0.255	−0.214	−0.183	0.585	0.841 *	0.518	−0.910 *	−0.401	−0.906 *	0.064
	*p*	0.626	0.528	0.729	0.223	0.036	0.292	0.012	0.431	0.013	0.904
3 + 4 MHIPP	r	−0.294	−0.137	−0.407	−0.033	0.422	0.332	−0.693	−0.259	−0.580	0.129
	*p*	0.571	0.689	0.423	0.950	0.404	0.520	0.127	0.620	0.228	0.808
SBMA	r	0.641	−0.169	0.544	0.017	−0.146	−0.566	0.195	0.218	0.151	0.305
	*p*	0.170	0.619	0.265	0.974	0.783	0.241	0.711	0.679	0.776	0.557

Bold indicates statistically significant values MA—mandelic acid; PGA—phenylglyoxylic acid; SPMA—S-phenylmercapturic acid; 2-MHIPP—2-methylhippuric acid, 3 + 4 MHIPP—3 and 4-methylhippuric acid; SBMA—S-benzyl mercapturic acid; MN—micronucleus; NB—nuclear buds; BIN—binucleated cells; KL—karyolytic cells; CC—condensed chromatin; TM—tail moment; TL—tail length.** The correlation is significant at 0.01—two tails; * The correlation is significant at 0.05—two tails.

**Table 6:** Pearson’s correlation analysis between (VOCs) and biomarkers of effect in the spray workers.

Biomarkers		Ethyl Acetate mg/m^3^	Toluene mg/m^3^	n-Butyl Acetate mg/m^3^	Ethylbenzene mg/m^3^	p-Xylene mg/m^3^	o-Xylene mg/m^3^	m-Xylene mg/m^3^
MN‰	r	0.146	0.006	−0.450	−0.248	−0.270	−0.350	−0.272
	*p*	0.782	0.991	0.370	0.636	0.604	0.497	0.602
NB‰	r	0.110	−0.187	−0.011	−0.211	−0.194	−0.132	−0.240
	*p*	0.836	0.723	0.984	0.688	0.713	0.803	0.647
MN+NB‰	r	0.150	−0.081	−0.245	−0.226	−0.230	−0.243	−0.255
	*p*	0.777	0.879	0.639	0.666	0.660	0.643	0.626
KL‰	r	0.405	0.279	0.835 *	0.710	0.781	0.937 **	0.721
	*p*	0.426	0.592	0.039	0.114	0.067	0.006	0.106
BIN‰	r	0.344	0.042	0.713	0.397	0.470	0.657	0.389
	*p*	0.504	0.938	0.112	0.435	0.346	0.157	0.446
CC‰	r	0.042	0.069	0.701	0.445	0.495	0.632	0.466
	*p*	0.936	0.896	0.121	0.377	0.319	0.178	0.351
TM	r	−0.369	−0.369	−0.716	−0.686	−0.773	−0.892 *	−0.719
	*p*	0.472	0.471	0.109	0.133	0.072	0.017	0.107
TL	r	−0.583	−0.496	−0.557	−0.714	−0.794	−0.872 *	−0.740
	*p*	0.225	0.317	0.251	0.111	0.059	0.024	0.093
Tail DNA%	r	0.199	0.200	−0.635	−0.076	−0.163	−0.352	−0.101
	*p*	0.705	0.704	0.176	0.887	0.758	0.494	0.849
Fpg sites Tail DNA%	r	−0.040	−0.038	−0.096	−0.171	−0.130	−0.119	−0.159
	*p*	0.940	0.942	0.856	0.746	0.805	0.822	0.764

Bold indicates statistically significant values. MN—micronucleus; NB—nuclear buds; BIN—binucleated cells; KL—karyolytic cells; CC—condensed chromatin; TM—tail moment; TL—tail length. * The correlation is significant at 0.05—two tails; ** The correlation is significant at 0.01—two tails.

**Table 7:** Oxidative stress and inflammation.

Subjects	Urinary Oxidized Nucleic Acid–Base µg/g Creatinine			Serum Cytokine		
	8-oxoGua	8-oxodGuo	8-oxoGuo	IL-6 pg/mL	IL-8 pg/mL	TNFα pg/mL
	Mean ± SD	Mean ± SD	Mean ± SD	concentration for subject with value > LOD n. subjects with values > LOD (%)		
Roller Painters N = 11	9.97 ± 12.26 *	5.23 ± 1.57 ^##^	14.51 ± 4.10 *	2.94 (subject 4)	271.94 (subject 10)	8.32 (subject 4)
					9.87 (subject 11)	4.62 (subject 8)
				1/11 (9.1%)	2/11 (18.2%)	2/11 (18.2%)
Spray painters N = 6	13.47 ± 17.01	6.09 ± 2.46 ^#^	19.10 ± 8.35 *	-	231.62 (subject 5)	-
				0/6 (0%)	1/6 (16.7%)	0/6 (0%)
All Painters N = 17	11.21 ± 13.69 °°	5.53 ± 1.90 ^	16.13 ± 6.12 °°	1/17 (0.6%)	3/17 (17.6%)	2/17 (11.7%)
Controls N = 18	41.86 ± 32.00	14.55 ± 7.40	9.05 ± 4.03		50.26 (subject 14)	4.85 (subject 18)
					3.71 (subject 18)	
				0/18 (0%)	2/18 (11.1%)	1/18 (5.5%)

LOD–limit of detection; IL-6 LOD = 0.92 pg/mL; IL-8 LOD = 2.00 pg/mL; TNFα LOD = 2.3 pg/mL. 8-oxoGua—8-oxo-7,8-dihydroguanine; 8-oxodGuo—8-oxo-7,8-dihydro-2′-deoxyguanosine; 8-oxoGuo—8-oxo-7,8-dihydroguanosine. Data relative to 8-oxoGua, 8-oxodGuo and 8-oxoGuo of painters are previously published in references [20,23]. * Kruskal–Wallis test. Multiple comparison: Dunn’s procedure adjusted *p*-value < 0.05 vs controls; °° Mann–Whitney *p*-value < 0.001 vs. controls; ^##^ ANOVA. Multiple comparison: T3—Dunnett ^##^ *p*-value < 0.001 vs. controls, ^#^ *p*-value < 0.05 vs controls; ^ T—Student’s test *p*-value < 0.001 vs. controls.

## Data Availability

The data presented in this study are available within this article. Further inquiries may be directed to the authors.
